# Systematic review of undeclared prohibited substances and pharmacological adulterants in dietary supplements: prevalence, detection, and risks in sport

**DOI:** 10.3389/fspor.2026.1740663

**Published:** 2026-03-13

**Authors:** Nasser Al-Saad, Shaikha Aljaal, Jawaher Alessa, Malavika E. Santhosh

**Affiliations:** 1 Qatar Anti-Doping Agency, Doha, Qatar; 2 Educational Research Center, College of Education, Qatar University, Doha, Qatar

**Keywords:** dietary supplements, doping, sports supplements, systematic review, WADA

## Abstract

**Introduction:**

The proliferation of dietary supplement use among athletes has heightened concerns over inadvertent doping due to contamination with substances prohibited by the World Anti-Doping Agency (WADA) and other undeclared pharmacological adulterants. This systematic review synthesizes empirical evidence on the prevalence, detection, and risks associated with sport supplement contamination. The review framework encompassed both explicitly prohibited doping agents and potent metabolic adulterants that pose significant regulatory and health risks.

**Methods:**

Following PRISMA guidelines, 12,031 records were initially identified across Scopus, Web of Science, and SPORTDiscus, of which 44 studies met the inclusion criteria.

**Results:**

Findings indicate that approximately 9–15% of commercially available supplements tested in empirical studies were contaminated with prohibited substances and unapproved pharmacological agents, predominantly stimulants and anabolic agents. Contamination was most frequently identified in pre-workout, weight-loss, and muscle-building products. Liquid Chromatography-Mass Spectrometry (LC-MS) emerged as the most widely used analytical method, followed by Gas Chromatography-Mass Spectrometry (GC-MS) and hybrid spectroscopic techniques, enabling high-sensitivity detection across diverse substance classes, including structural analogs and emerging metabolic markers.The findings highlight a critical gap between product labeling and actual chemical composition, posing a persistent threat to athlete integrity and public health.

**Discussion:**

This review stresses the need for more stringent international manufacturing standards, expanded analytical screening protocols that account for emerging structural analogs, and enhanced athlete education to mitigate the risk of unintentional doping.

## 1 Introduction

The use of dietary supplements is deeply embedded in contemporary sport, spanning recreational, amateur, and elite athletic contexts. Athletes consume supplements with the expectation of enhanced performance, accelerated recovery, or improved nutritional balance (1, 2). However, the growth of this industry is accompanied by uneven regulation, inadequate quality control, and significant risks of contamination with substances prohibited by the World Anti-Doping Agency (WADA), as well as other undeclared pharmacological adulterants. Indeed, such contamination undermines the credibility of sport and places athletes under the threat of unintentional anti-doping rule violations (ADRVs), which can result in suspensions, reputational damage, and long-term consequences for athlete integrity (3, 4).

Despite the global scope of this issue, existing scholarship indicates that awareness of supplement contamination is limited, and regulatory approaches vary across regions (5, 6). International consensus statements and the 2026 WADA guidelines have sought to harmonize standards; however, disparities in oversight, education, and enforcement continue to expose athletes to preventable susceptibility (7). Moreover, the principle of zero-tolerance liability in anti-doping policy intensifies these challenges, as athletes are held accountable for substances ingested regardless of intent (8). This dilemma between personal responsibility and institutional shortcomings has cast supplement contamination as a public health concern and a contested arena for debates about fairness, accountability, and ethics in sport.

Thus, this systematic review addresses these challenges by investigating the prevalence and detection of both explicitly WADA 2026-listed doping agents and “catch-all” substances that occupy complex regulatory spaces (9). Furthermore, this study evaluates the presence of non-approved metabolic adulterants, which pose analogous risks to athlete health and legal standing.

This paper is structured as follows. The next section presents a review of the literature on the global anti-doping regulatory landscape, supplement contamination, and critical gaps. The methodology section outlines the systematic review design, including the search strategy, inclusion and exclusion criteria, and analytical approach in accordance with PRISMA guidelines. The results section reports the prevalence and types of prohibited substances and pharmacological adulterants identified, the categories of products most frequently affected, and the analytical techniques used for detection. The discussion interprets these findings in relation to athlete integrity and the WADA 2026 regulatory landscape, highlighting implications for anti-doping policy and education. The paper concludes by summarizing the key contributions of the review, acknowledging its limitations, and outlining directions for future research.

## 2 Literature review

The intersection of supplement use and anti-doping efforts in sport has increasingly examined in its intersection with anti-doping regulation, with particular attention to the risks of inadvertent doping, inconsistencies in regulatory oversight, and the implications of policy design across diverse sporting contexts (10–12). The market realities of many countries introduce additional complexities into anti-doping governance. In particular, limited regulation of supplement markets, variability in educational provision, and uneven anti-doping infrastructures may increase athletes' exposure to inadvertent doping risks while compliance obligations continue to be placed mainly on individuals (10, 13).

### 2.1 Global supplement use and the anti-doping regulatory landscape

The prevalence of supplement use among athletes is extensively documented, with usage spanning from recreational participants to elite professionals who utilize products for training support, recovery, and performance enhancement (14–16). Despite their popularity, studies reveal widespread misinformation regarding safety, compounded by inconsistent regulatory oversight across jurisdictions (12). These variations highlight the global nature of supplement use, where international policies, specifically the 2026 WADA Prohibited List, interact with diverse local market dynamics and cultural perceptions of risk.

The 2026 WADA Prohibited List defines the regulatory boundaries of anti-doping by categorizing banned substances into classes such as anabolic agents (S1), hormone and metabolic modulators (S4), and stimulants (S6). Crucially, these categories include “catch-all” clauses for substances with a similar chemical structure or similar biological effect(s), thereby encompassing emerging structural analogs and unapproved pharmacological agents (S0) that may not be explicitly named/banned (9, 17).

WADA seeks to enforce globally harmonized norms through the World Anti-Doping Code. However, the translation of these norms into practice depends heavily on national anti-doping organizations (NADOs) and their capacity, resources, and consistency with local cultural norms (6, 18). Power asymmetries are evident: while well-resourced NADOs in Europe and North America implement extensive testing, counterparts in the Global South often face financial constraints that limit their surveillance of local supplement markets (19–21). This results in uneven risk exposure, as athletes in under-resourced contexts may lack access to third-party vetted products or credible education, relying instead on peer advice or informal online sources (13, 22). Under the principle of strict liability, this systemic failure places a disproportionate burden on the individual athlete, for whom a contaminated supplement represents not just a biomedical risk, but a critical governance and integrity challenge (23).

### 2.2 Doping, contamination, and the athlete integrity framework

Beyond the biomedical risks, supplement contamination challenges the “athlete integrity” framework, a normative model that shifts focus from punitive enforcement to moral reasoning and transparency (24, 25). The concept of “athlete integrity” has gained traction as a normative framework with values such as fair play, responsibility, and transparency. This reframing moves beyond the binary of “clean vs. dirty” sport, acknowledging the complex psychological and contextual pressures athletes face.

However, the presence of undeclared pharmacological agents creates a “situational vulnerability” where athlete integrity is eroded by systemic failures rather than individual intent (26). As highlighted by recent analyses, these risks individualize responsibility through the principle of strict liability, failing to account for the structural inequities faced by athletes in lower-resource regions who lack access to third-party vetted products (27). For these athletes, ethical reasoning alone is insufficient unless supported by institutional accountability and the elimination of undeclared contaminants from the global supply chain.

### 2.3 Policy gaps, cultural and religious dimensions in anti-doping: A critical gap

A significant gap in existing literature pertains to how cultural and religious perspectives influence supplement use and anti-doping compliance. While localized insights exist for Turkey, Iran, and Malaysia (28), there is a dearth of systematic research on how Arab-Islamic societies conceptualize integrity, bodily discipline, or rule compliance within sport (29, 30). In these contexts, athletes often frame integrity through communal expectations of purity and reputation (31, 32). Concepts such as *‘Ird* (honor) intensify the stigma of rule violations, shaping self-regulation differently from the secularized individualist ethics prevalent in European anti-doping narratives (92) or the spiritual body cultures of Latin America (33). Understanding these culturally embedded factors, including honor, shame, and collective identity, is essential for aligning regulatory frameworks with athletes lived contexts (34–37).

Beyond cultural factors, the practical implementation of WADA policies reveals stark regional disparities (38). Variations in funding, legal infrastructure, and political environments often result in the uneven adoption of testing and education (17, 39–41). These “capacity asymmetries” regarding NADO independence and Code compliance highlight why many Global South countries, including parts of the Arab-Islamic world, remain under-resourced and fragmented (21, 42, 43). Consequently, there is an urgent need for “smart regulation” that integrates strict enforcement with culturally congruent education and stakeholder collaboration to mitigate the risk of inadvertent doping (44–46).

### 2.4 Analytical evolution: From targeted testing to non-targeted screening

The detection of undeclared substances in dietary supplements represents a moving target for analytical chemists and regulatory bodies. Historically, screening relied on targeted assays, which could only identify substances explicitly listed by name in the Prohibited List. However, the emergence of “designer” structural analogs and unapproved pharmacological agents has necessitated a shift toward non-targeted screening and High-Resolution Mass Spectrometry (HRMS**)** (12, 15). Current scholarship identifies Liquid Chromatography-Mass Spectrometry (LC-MS) as the gold standard for detecting these contaminants due to its high sensitivity and ability to characterize unknown chemical structures without prior reference standards (9). This technical evolution is critical for enforcing WADA's “catch-all clauses”, as traditional gas chromatography methods often lack the resolution required for complex botanical matrices or novel synthetic stimulants (47). Consequently, the disparity in access to such advanced instrumentation between well-resourced and under-resourced NADOs remains a primary technical barrier to global anti-doping harmonization (17, 21).

## 3 Methodology

**Figure 1:**
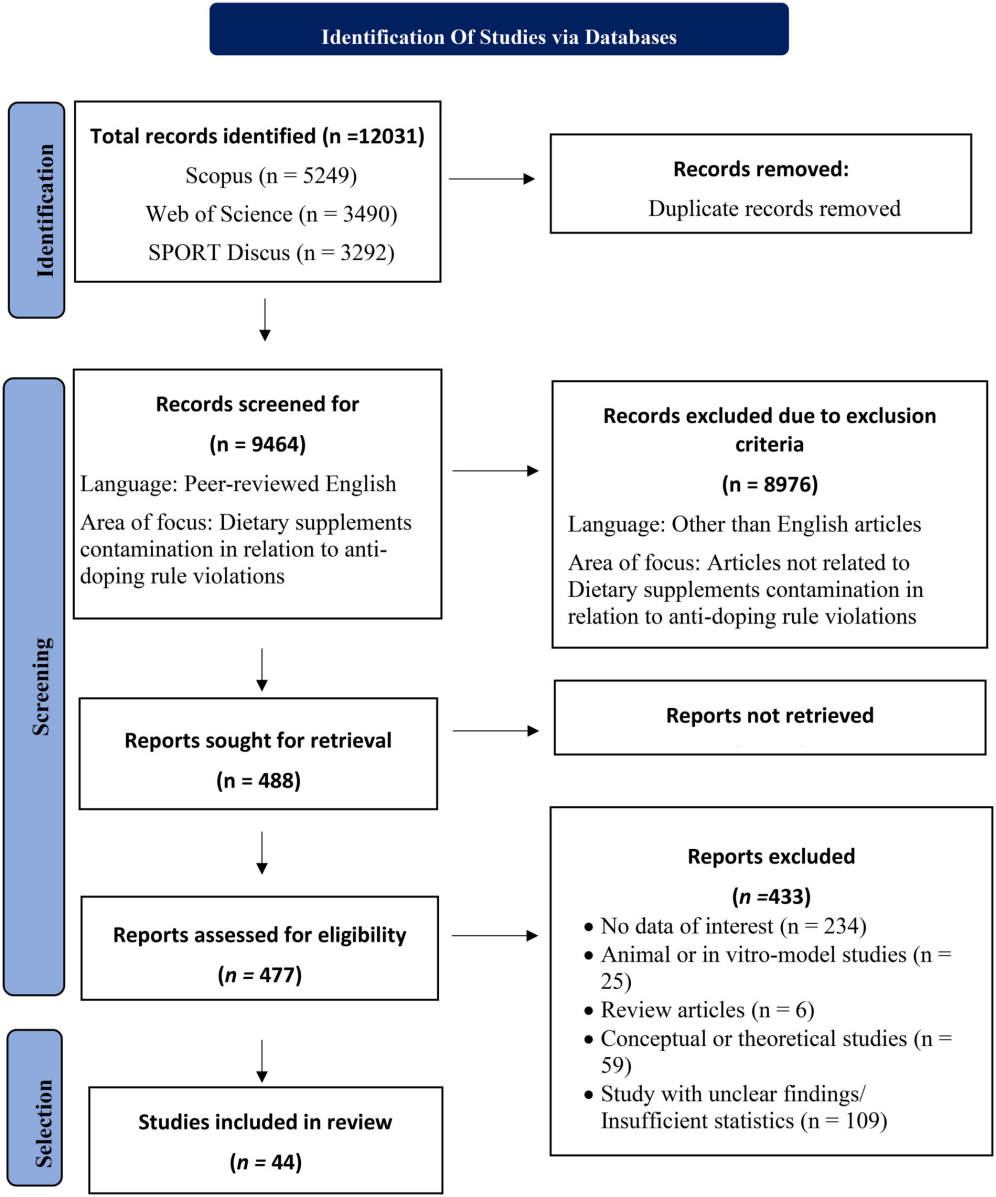
PRISMA 2020 flow diagram revealing the inclusion-exclusion criteria for studies selected for this systematic review (48).

This systematic review adhered to the PRISMA guidelines to ensure methodological transparency, reproducibility, and minimization of bias throughout the review process (48). Following the PRISMA model, the study was structured into three sequential phases (identification, screening, and selection), each designed to progressively refine the evidence base (48). Figure 1 illustrates the application of the PRISMA methodology across three distinct phases.

The scope of this systematic review is to showcase the global supplement landscape, focusing on the prevalence and detection of undeclared pharmacological agents in sport supplements. The primary purpose is to identify the specific classes of WADA-prohibited substances and non-approved adulterants, including “catch-all” stimulants and metabolic modulators in supplement categories. Furthermore, the study evaluates the efficacy of current analytical methodologies in characterizing these contaminants. In doing so, the study aims to generate a comprehensive and context-sensitive understanding of supplement contamination, while highlighting implications for policy, education, and athlete protection. The following are the research questions explored in this systematic review:

1. Which WADA-prohibited substances and other pharmacologically active adulterants have been detected in dietary supplements commonly used in sport?
2. In which dietary supplement categories are these prohibited substances and adulterants most frequently found
3. Which analytical techniques are most commonly used to detect undeclared substances in dietary supplements, and what categories of pharmacological agents do they identify?

### 3.1 Search strategy

A comprehensive search strategy was employed to identify relevant studies for this systematic review. Literature searches were conducted across three major scientific databases: Scopus, Web of Science, and SPORTDiscus. The search utilized a combination of Boolean keywords, including: (“anti-doping” OR “doping” OR “unintentional doping” OR “inadvertent doping”) AND (“supplement” OR “dietary supplement” OR “nutritional supplement” OR “undeclared substance” OR “supplement contamination” OR “nutraceutical” OR “ergogenic aid” OR “performance-enhancing drugs” OR “steroids” OR “prohibited” OR “stimulants” OR “banned substance” OR “analog” OR “adulterants”). These terms were selected to capture studies related to the presence of WADA-prohibited substances or non-approved adulterants in sports dietary supplements.

### 3.2 Inclusion and exclusion criteria

This systematic review considered original empirical studies published between 2010 and March 2025, written in English. Review articles, conceptual papers, book chapters, reports, and conference proceedings were excluded. The review focused on the presence of undeclared prohibited substances and pharmacological adulterants in dietary supplements that pose a risk to athletes and consumers. To ensure a comprehensive overview of the risk landscape, the study included: (1) WADA-Prohibited Substances: Substances explicitly listed by WADA listed or those falling under “catch-all” clauses; (2) Pharmacologically Active Adulterants: those which are not explicitly named on the 2026 WADA list, but are potent metabolic agents frequently used as an undeclared adulterant in supplements and pose similar health and regulatory risks; (3) Other Not-Always-Prohibited Substances/ markers of high risk contaminations: Other substances of conditional or contextual anti-doping relevance, included to reflect documented cases of multi-substance contamination where non-prohibited compounds are analytically detected alongside prohibited substances and may contribute to regulatory ambiguity or unintentional doping risk. Therefore, the scope includes both substances explicitly named in the 2026 WADA Prohibited List and emerging structural analogs captured under “catch-all” regulatory clauses (S0 and S6).

Studies centered on athletes' doping behaviors, supplement usage patterns, intervention programs, general health effects of supplements, or those lacking evidence of contamination or anti-doping violations were excluded. Studies focused solely on consumer behavior or general health effects without analytical screening for undeclared/prohibited components were excluded. A total of 46 studies met the inclusion criteria and were selected for the review.

### 3.3 Data extraction

Data extraction for this review was performed independently by two reviewers (researchers) using a customized extraction form developed in accordance with the best practices in data extraction (20). To ensure accuracy and consistency, a cross-checking process was implemented. The extracted data comprised general study details, including author names, publication year, journal, and country of study. Additionally, it incorporated key aspects relevant to the research scope, such as participant characteristics (school level and sample size), study settings, objectives, methodologies, theoretical frameworks, instruments used, and the relationships investigated.

### 3.4 Reliability test

Data extraction for this review was carried out independently by two reviewers (the authors) using a tailored extraction form (Excel sheet), developed in alignment with established best practices in systematic reviews. To maintain accuracy and consistency, a thorough cross-checking process was conducted, allowing discrepancies to be identified and resolved collaboratively between the reviewers during screening and data extraction. The extracted information included study details such as study name, authors, year of publication, the country in which the study was conducted, undeclared substances in dietary supplements, mode of detection, and key findings. This structured approach ensured that all relevant aspects were consistently documented for analysis and synthesis.

Kappa coefficient (test of reliability) yielded a value of 0.753 (*p* < 0.001), indicating substantial agreement between the reviewers. Any discrepancies in the selection process were resolved through discussion.

## 4 Results

### 4.1 Descriptive findings

**Figure 2:**
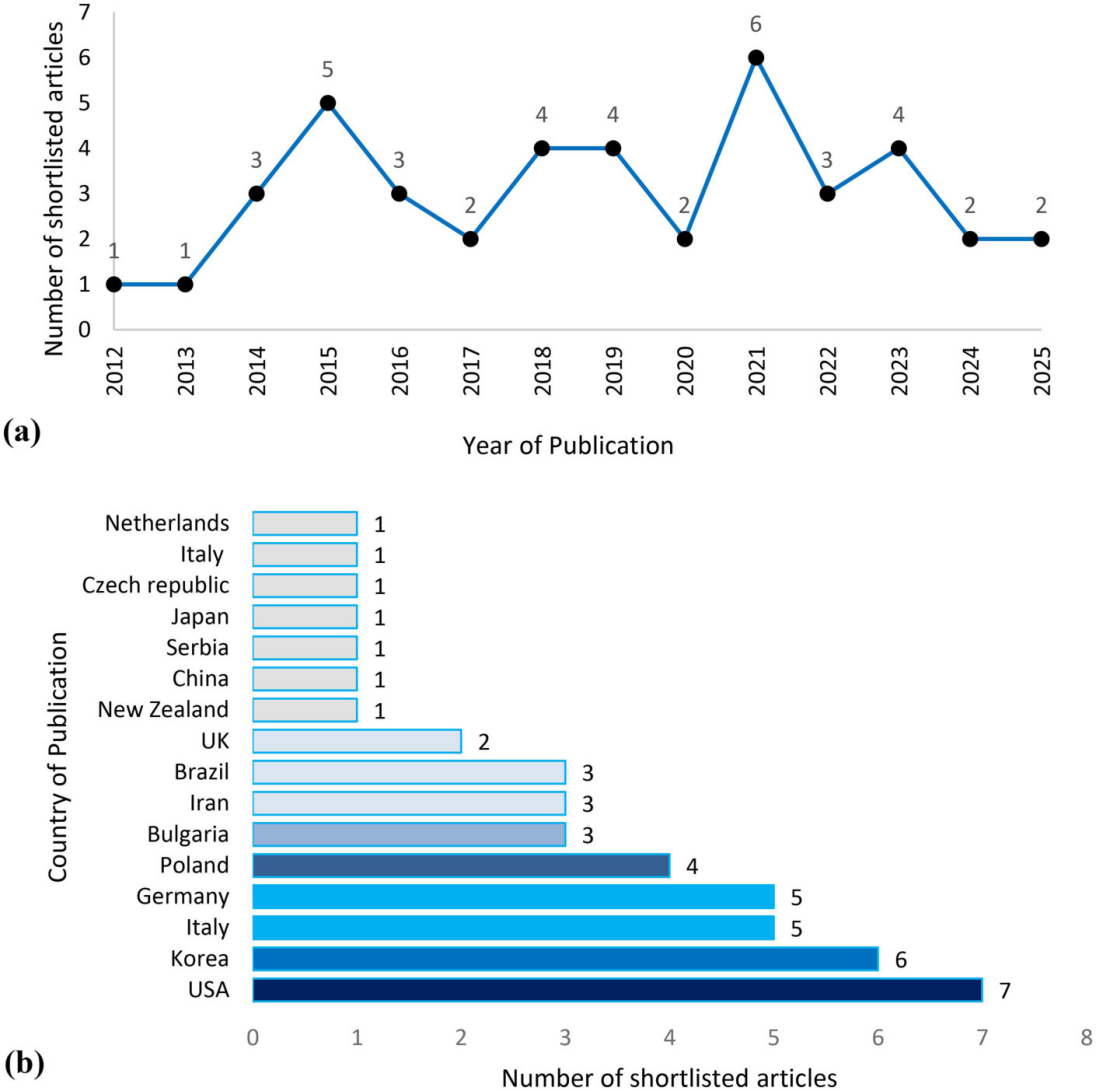
Graphical representation of the shortlisted articles by **(a)** year and **(b)** country of publication.

Figure 2 illustrates the distribution of the shortlisted articles by (a) year and (b) country of publication. It should be noted that much of the descriptive evidence on supplement contamination derives from studies conducted in general or multinational athlete populations. Accordingly, general population findings are presented here to establish baseline patterns and structural conditions that frame subsequent discussion of culturally and ethically specific dynamics.

The research on dietary supplement contamination appears to be highly scattered over time, showing no consistent trend or pattern in publication frequency. In terms of geographic origin, the United States leads with the highest number of studies (*n* = 7) reporting contamination of dietary supplements in relation to anti-doping violations. This is followed closely by Korea (*n* = 6). Additionally, Italy, and Germany each contributed five studies, indicating a notable level of research interest in these countries as well. This geographical dispersion highlights the global concern surrounding unintentional doping through contaminated supplements, though research efforts remain unevenly distributed.

*RQ 1: Which WADA-prohibited substances and other pharmacologically active adulterants have been detected in dietary supplements commonly used in sport?*

**Figure 3:**
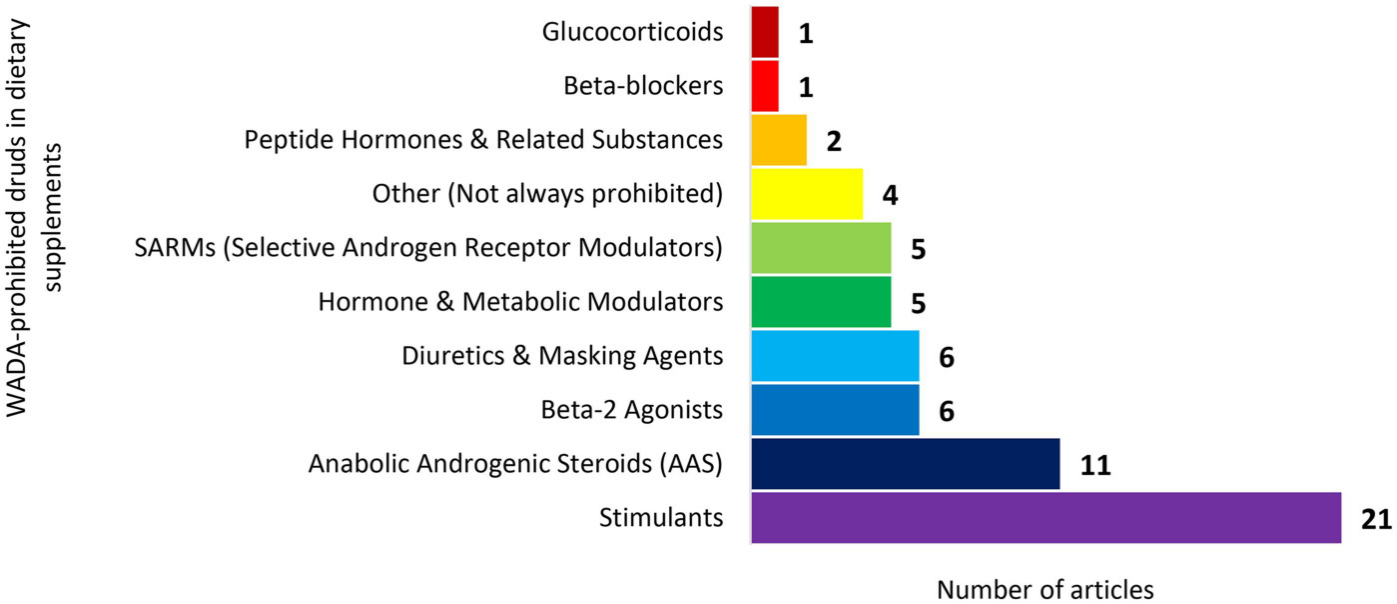
The number of shortlisted articles by category of dietary supplement contaminated with WADA-prohibited substances and non-approved adulterants.

The analysis of dietary supplements commonly used in sport revealed the presence of a wide range of WADA-prohibited substances and non-approved adulterants, spanning multiple pharmacological and chemical classes. The most commonly detected are the stimulants (*n* = 21), which represent a chemically diverse group of compounds that primarily act on the central nervous system to increase alertness, reduce fatigue, and enhance physical performance (49–52). These include synthetic derivatives of amphetamines (e.g., DMAA, NNDMPPA, N, α-DEPEA, BMPEA, 1,3-DMBA, Octodrine, Phenpromethamine), phenethylamine analogs (*Β*-Methylphenethylamine, Phenethylamine, Oxilofrine, N-Methyltyramine, Octopamine, Hordenine), thermogenic and appetite-suppressing agents (Ephedrine, Sibutramine, Phentermine, Phendimetrazine, Fenfluramine, Mephentermine, Fen-Canfamine), etc. (refer to Table 1 and Figure 3).

**Table 1:** Summary of undeclared WADA-prohibited substances and pharmacological adulterants detected in dietary supplements.

WADA Category	Example Substances	Articles
Anabolic Agents: Anabolic-Androgenic Steroids (AAS)	DHEA, Methyl-1-testosterone, Androstenedione, Methandienone, Mesterolone, Oxandrolone, Stanozolol, Nandrolone (propionate/decanoate), Trenbolone (acetate/enanthate), Boldenone undecylenate, Testosterone (various esters), Clostebol, 19-norandrostenedione, 19-nortestosterone, Mandol, Androstane derivatives	(52–62)
Anabolic Agents: Selective Androgen Receptor Modulators (SARMs)	Andarine, Ostarine, LGD-4033, RAD-140, YK-11, S23, ACP-105, LGD-3303, SR-9009, GW-501516, Ligandrol, Testolone	(63–67)
Hormone & Metabolic Modulators	Tiratricol, Arimistane	(56, 58, 67–69)
Peptide Hormones & Related Substances	Growth Hormone Releasing Peptide (GHRP-2), Ibutamoren (MK-677)	(67, 70)
Beta-2 Agonists	Higenamine	(50, 52, 58, 61, 71, 72)
Beta-blockers	Propranolol, Atenolol, Metoprolol, Nadolol, Bisoprolol	(52)
Stimulants (including “catch all” clause)	DMAA (1,3- and 1,4-), N, N-dimethyl-2-phenylpropan-1-amine (NNDMPPA), N,*α*-DEPEA, β-methylphenethylamine, BMPEA, 1,3-DMBA, Octodrine, Deterenol, Phenpromethamine, Oxilofrine, Phenethylamine, N-methyltyramine, Octopamine, Hordenine, Ephedrine, Sibutramine, Phentermine, Phendimetrazine, Fenfluramine, Mephentermine, Fen-Canfamine	(49–52, 58, 61, 67, 71, 73–85)
Diuretics & Masking Agents	Hydrochlorothiazide (HCTZ), Chlorazanil, Bendroflumethiazide, Bumetanide, Amiloride, Acetazolamide	(50, 56, 58, 61, 86, 87)
Glucocorticoids	Dexamethasone	(76)
Emerging risks and regulatory markers or non-prohibited pharmacological adulterants	Pseudoephedrine (threshold substance), Fluoxetine, PDE5 inhibitors (Sildenafil, Vardenafil, Tadalafil), Designer steroids (Hemapolin)	(51, 63, 76, 81)

Substances are categorized by pharmacological class, including those explicitly listed by WADA and those included via “catch-all” clauses for structural/biological similarity (e.g., hordenine), while non-prohibited substances (e.g., PDE5 inhibitors) and metabolic adulterants (e.g., tiratricol) are included as markers of high-risk, undeclared contamination.

**Figure 4:**
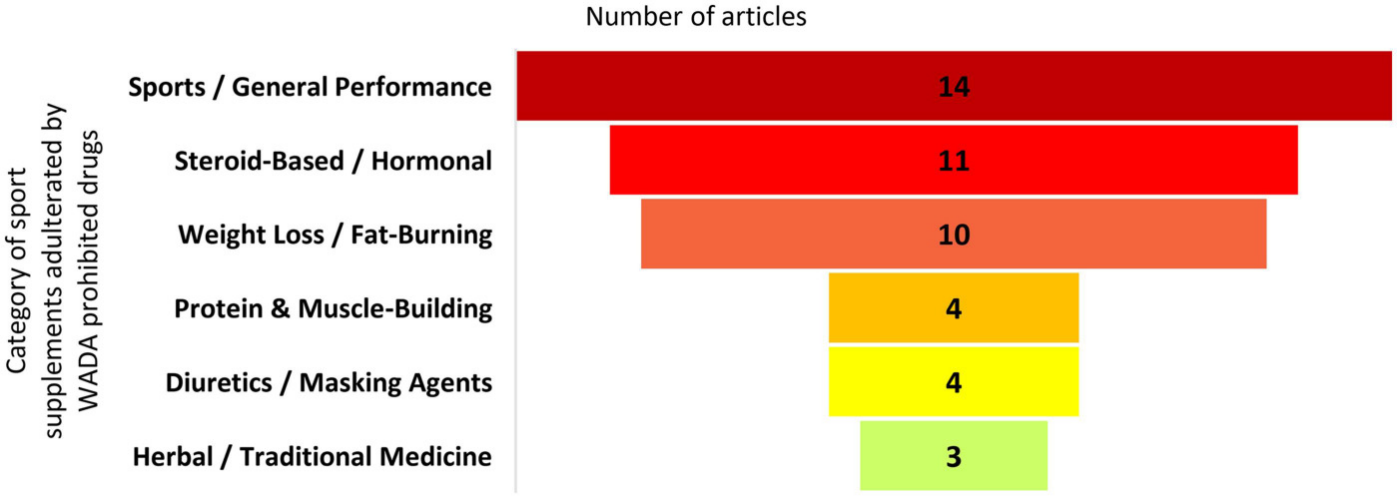
Number of articles demonstrating the category of WADA-prohibited and non-approved adulterants in dietary supplements.

The next widely reported contaminant in dietary supplements is the Anabolic Agents, including Anabolic Androgenic steroids (AAS) and Selective Androgen Receptor Modulators (SARMs) (*n* = 11 and *n* = 5, respectively. Refer to Table 1 & Figure 4). AAS mimics the effects of endogenous androgens, promoting increased muscle mass, strength, power, and accelerated recovery, making them attractive for enhancing athletic performance and body composition. Hormone and metabolic modulators that alter hormonal balance or metabolic pathways to enhance performance or body composition were also identified in several supplements (*n* = 5) (56, 58, 67, 68, 70). Similarly, SARMs were detected in multiple studies (*n* = 5) (63, 64, 66, 67, 76). SARMs are designed to selectively stimulate androgen receptors, promoting anabolic effects in muscle and bone while minimizing androgenic side effects.

Among beta-2 agonists, Higenamine was the most frequently reported adulterant (*n* = 6), known for its bronchodilatory and stimulant effects, which can enhance endurance and fat metabolism (50, 52, 58, 61, 72, 73). Diuretics and masking agents, including hydrochlorothiazide, bendroflumethiazide, bumetanide, amiloride, and acetazolamide, were also reported (*n* = 6) (50, 51, 56, 58, 86, 87). These compounds can increase urine output, mask the presence of other prohibited substances, and facilitate rapid weight loss.

Likewise, peptide hormones and related substances, such as growth hormone-releasing peptide (GHRP-2) and ibutamoren (MK-677), were reported (*n* = 2), which stimulate endogenous hormone release (67, 70). Beta-blockers, including propranolol, atenolol, metoprolol, and nadolol, were identified less commonly (*n* = 1); these substances act to reduce heart rate and tremors, and are sometimes misused in precision sports requiring steady hands (52). Similarly, Glucocorticoids, represented by dexamethasone, appeared less frequently (*n* = 1) and are used for their anti-inflammatory and immunosuppressive effects (76). Findings illustrate that dietary supplements commonly used in sport may contain multiple classes of WADA-prohibited substances and non-approved adulterants, highlighting the potential risk of inadvertent anti-doping violations for athletes.

***RQ 2:*** *In which dietary supplement categories are these prohibited substances and adulterants most frequently found?*

Analysis of the literature (Table 2) indicates that the adulteration of sport supplements with WADA-prohibited substances and non-approved adulterants is widespread, with certain product categories being particularly susceptible. The sports and general performance supplements category emerged as the most frequently adulterated category (*n* = 14; Figure 3), encompassing pre-workout formulas, energy boosters, and general performance enhancers (70, 75, 81). These products are often spiked with stimulants, anabolic agents, or other performance-enhancing compounds (Table 1) to provide rapid effects, sometimes without disclosure on the label (50, 67, 85).

**Table 2:** Categories of sport supplements commonly adulterated with WADA-prohibited substances and non-approved adulterants.

Category	Supplement/Product Description	Articles
Protein & Muscle-Building	OXPUMP Pre-Training Formula, Fruit Punch, Body-building supplements, Pre-workout supplement, Whey Protein Food Supplement (WPFS)	(58, 73, 83, 84)
Weight Loss/Fat-Burning	Weight loss supplements, herbal weight loss tea, weight loss pills	(51, 56, 68, 71, 72, 74, 80, 85, 88, 89)
Steroid-Based/Hormonal	Steroid-based dietary supplement	(53–55, 57, 59, 60, 62, 64–66, 76)
Herbal/Traditional Medicine	Geraniaceae, Geranium oil, dietary supplements, traditional Chinese medicines, herbal tea	(49, 69, 80)
Sports/General Performance	General sport dietary supplements, energy boosters, pre-workout formulas, and general performance enhancers	(50, 52, 61, 63, 67, 70, 75–79, 81, 82, 85)
Diuretics/Masking Agents	Non-steroidal anti-inflammatory capsule, anti-malarial drug	(56, 69, 86, 87)

Steroid-based or hormonal supplements (*n* = 11) are explicitly marketed to enhance muscle growth, strength, or recovery. These products are frequently adulterated with anabolic-androgenic steroids or prohormones, directly introducing substances banned by WADA and posing serious health risks (53, 54, 57). Weight loss and fat-burning supplements (*n* = 10) include dietary pills, herbal teas, and fat-reducing formulas, which are often adulterated with stimulant compounds such as sibutramine or other amphetamine analogues to enhance metabolic rate and suppress appetite, which not only contravenes anti-doping regulations but also endangers cardiovascular health (68, 80, 88).

Protein and muscle-building supplements (*n* = 4), such as whey protein powders and pre-training formulas, have also been found to contain undeclared stimulants, prohormones, or anabolic agents (58, 73, 83, 84). Herbal and traditional medicines (*n* = 3), including traditional Chinese medicines, herbal teas, or plant-based extracts, are occasionally adulterated with synthetic steroids or stimulants (i.e., methylhexanamine, derived from the Geraniaceae family) to enhance perceived efficacy (49, 69, 80). Finally, diuretics and masking agents (*n* = 4), including non-steroidal anti-inflammatory capsules or anti-malarial drugs, are added to some supplements to mask the presence of prohibited compounds in doping tests, presenting additional risks for athletes (56, 69, 86, 87).

Overall, these findings indicate that adulteration occurs across a wide range of supplement categories, but is particularly pronounced in performance-enhancing, steroid-based, and weight-loss products. Practice not only jeopardizes fair competition in sports but also poses significant health risks to consumers, highlighting the need for strict regulatory oversight, third-party testing, and increased athlete and consumer awareness.

***RQ 3:*** *Which analytical techniques are most commonly used to detect undeclared substances in dietary supplements, and what categories of pharmacological agents do they identify?*

Table 3 summarizes the analytical techniques employed to detect WADA-prohibited adulterants and non-approved adulterants in dietary supplements and the categories of substances they identify. Among the reviewed studies, Liquid Chromatography-Mass Spectrometry (LC-MS) emerges as the most widely utilized method (*n* = 28; refer to Table 3). This category encompasses a range of approaches, including High-Performance Liquid Chromatography–Mass Spectrometry (HPLC-MS), Liquid Chromatography–Tandem Mass Spectrometry (LC-MS/MS), Ultra-Performance Liquid Chromatography–Mass Spectrometry (UPLC-MS/MS), Ultra-High-Performance Liquid Chromatography–Mass Spectrometry (UHPLC-MS/MS), Liquid Chromatography–Electrospray Ionization–Tandem Mass Spectrometry (LC–ESI–MS/MS), Liquid Chromatography–Triple Quadrupole Mass Spectrometry (LC–QQQ-MS), Liquid Chromatography–Quadrupole Time-of-Flight Mass Spectrometry (LC–QTOF-MS), High-Resolution/Accurate-Mass LC-MS (LC-HRMS/LC-HRAM-MS), Orbitrap-based LC-MS systems (UHPLC-Q-Orbitrap, LTQ-Orbitrap, Q-Exactive Orbitrap HRMS), and Hydrophilic Interaction Liquid Chromatography–MS/MS (HILIC-MS/MS). These methods have been applied to detect a broad spectrum of WADA-prohibited substances, including stimulants, hormone and metabolic modulators, anabolic androgenic steroids (AAS), selective androgen receptor modulators (SARMs), peptide hormones and related substances, diuretics and masking agents, beta-2 agonists, and glucocorticoids.

**Table 3:** Analytical techniques used to detect WADA-prohibited adulterants in dietary supplements and their target substance categories.

Detection Method	Category of Substance Detected	Shortlisted Articles
Liquid Chromatography–Mass Spectrometry (LC-MS) (*n* = 28)	Stimulants, Hormone & Metabolic Modulators, Anabolic AAS, SARMs, Peptide Hormones & Related Substances, Diuretics & Masking Agents, Beta-2 Agonists, Glucocorticoids.	(49–54, 56–58, 60, 61, 65–74, 76, 80, 81, 84, 86, 87, 90)
Gas Chromatography–Mass Spectrometry (GC-MS) (*n* = 6)	AAS, Stimulants, Beta-2 agonists	(52, 53, 77, 78, 82, 83)
Chromatography with Other Detectors (*n* = 5)	AAS, SARMs, Hormone & Metabolic Modulators	(53, 59, 62, 65, 68)
Spectroscopy Methods (*n* = 4)	AAS, SARMs, Stimulants	(53, 64, 79, 85)
Advanced Hybrid & Specialized Techniques (*n* = 4)	AAS, SARMs, Stimulants	(56, 64, 69, 75)
Bioassay-/ Immunoassay-based Methods (*n* = 2)	AAS, SARMs	(55, 63)

Not all studies applied identification criteria compliant with WADA TD IDCR.

Gas Chromatography-Mass Spectrometry (GC-MS) was the next most frequently reported technique (*n* = 6), primarily detecting AAS, stimulants, and beta-2 agonists (52, 53, 77, 78, 82, 83). Chromatography-based approaches in conjugation with detectors were also used for the detection of AAS, SARMs, and hormone and metabolic modulators (53, 59, 62, 65, 68). These included High-Performance Liquid Chromatography-Diode Array Detector (HPLC-DAD), Ultra-High-Performance Liquid Chromatography–Photodiode Array detector (UHPLC-PDA), Liquid Chromatography with Ultraviolet Detection (LC/UV), Liquid Chromatography–Visible Spectroscopy (LC-Vis), and High-Performance Thin Layer Chromatography (HPTLC).

The Spectroscopy-based methods, including Nuclear Magnetic Resonance (NMR), Raman spectroscopy, and standalone Ultraviolet–Visible (UV-Vis) spectroscopy, were employed to identify AAS, SARMs, and stimulants (53, 64, 79, 85). Advanced hybrid and specialized techniques, such as Direct Analysis in Real Time Tandem Mass Spectrometry (DART-MS/MS), Isotope-Ratio Mass Spectrometry (IRMS, when not GC-linked), QuEChERS extraction followed by LC-MS/MS, Differential Scanning Calorimetry (DSC), Thermogravimetry (TGA), and x-ray Powder Diffraction (XRD), have also been applied to detect AAS, SARMs, and stimulants (56, 64, 69, 75).

## 5 Discussion

The evidence produced across the reviewed literature highlights a significant prevalence of undeclared prohibited substances and pharmacological adulterants in dietary supplements, particularly stimulants and AAS. Stimulants, such as DMAA, octodrine, and sibutramine, were the most frequently detected compounds, reflecting their widespread and illegal use for enhancing alertness, thermogenesis, and endurance (49, 71, 74). Similarly, AAS, including nandrolone derivatives and testosterone esters, were reported in multiple studies demonstrating their undeclared presence in bodybuilding products (53, 57).

Compared to stimulants, AAS carry more severe long-term health consequences, including cardiovascular and endocrine dysfunctions, but both groups pose a major threat for inadvertent anti-doping rule violations. Other substance classes, such as SARMs (e.g., Ostarine, RAD-140) and peptide hormones (e.g., GHRP-2, ibutamoren), though less frequently detected demonstrate the expanding effort of sport dietary supplement adulteration (63, 64). The findings indicate that while stimulants dominate in numerical frequency, the pharmacological diversity of WADA-prohibited contaminants underlines the complexity of the anti-doping challenge.

Analysis of product categories reveals that sports and general performance supplements were the most commonly adulterated, typically containing stimulants and anabolic compounds designed to deliver immediate and noticeable performance effects (50, 81). Weight-loss supplements constitute the second most adulterated category, with products often spiked with sibutramine or amphetamine analogues to accelerate fat metabolism and appetite suppression (68, 85). Steroid-based and muscle-building supplements also consistently contained undeclared anabolic steroids and prohormones, increasing risks of performance enhancement and adverse health outcomes (55, 60).

By contrast, herbal and traditional supplements were less frequently adulterated, but when spiked, they often contained potent stimulants such as methylhexanamine, concealed under natural plant derivatives (49, 80). This pattern illustrates that while mainstream performance and weight-related supplements dominate in prevalence, the intermittent contamination of “natural” supplements may pose even greater risk due to consumer misconceptions of safety.

Across the reviewed studies, LC-MS and its advanced variants (LC-MS/MS, LC-QTOF-MS, LC-HRMS) emerged as the gold standard, enabling high-sensitivity detection across nearly all WADA substance categories, including stimulants, AAS, SARMs, and diuretics (50, 60, 84). GC-MS was comparatively less common but remained particularly effective for volatile and thermally stable compounds, particularly stimulants, beta-2 agonists, and AAS (52, 83).

In contrast, chromatography paired with alternative detectors such as diode array, UV-visible spectroscopy was employed primarily for targeted detection of AAS, SARMs, and hormone modulators (62, 68). These methods are comparatively more accessible in resource-limited settings, and they offer narrower substance coverage and reduced sensitivity relative to MS-based approaches. More recently, advanced hybrid methods such as DART-MS/MS, IRMS, and XRD have expanded detection capabilities, especially for complex or novel adulterants (56, 64).

Finally, bioassay- and immunoassay-based methods (e.g., ELISA, LFIA, yeast androgen assays) were the least reported (*n* = 2) but provide functional insights into the biological activity of adulterants, which purely chemical analyses may overlook (55, 63). Overall, comparative evidence highlights that while LC-MS techniques dominate due to their wide applicability and reliability, complementary approaches are essential to keep pace with the evolving complexity of supplement adulteration.

Although the majority of studies included in this review focused on traditional categories of supplements such as pre-workouts, herbal weight-loss, and steroid-based products, recent evidence highlights an emerging category of nootropic or “brain doping” supplements that may also contain WADA-prohibited substances (91). This systematic review also highlights the prevalence/detection of substances such as Deterenol, Hordenine within dietary supplements (85). Although not explicitly named in the WADA Prohibited List, it was included in this review as a prohibited stimulant under the S6 category due to its structural similarity to prohibited phenethylamine derivatives, and documented occurrence as an undeclared ingredient in dietary supplements, which may present a potential anti-doping risk depending on context. This classification aligns with recent literature by Pokrywka et al. (9), who highlight that substances like hordenine are increasingly used in “brain doping” and dietary supplements to bypass specific regulations, despite their potential to trigger adverse analytical findings under the “similar biological effect.”

In the Australian online marketplace, 35% of sports supplements tested contained prohibited substances, predominantly naturally occurring stimulants, and a significant portion of these were unlabeled. These findings underscore the ongoing risk of inadvertent anti-doping rule violations and expand the spectrum of products that athletes must scrutinize.

It's noteworthy that most of the studies included in this review employed advanced analytical techniques such as LC-MS and GC-MS for the detection of WADA-prohibited substances and non-approved adulterants in dietary supplements; however, a significant variability was observed in the analytical identification criteria applied across studies (52, 53, 68, 69, 71, 73, 86). Several investigations reported the presence of prohibited compounds based on analytical confirmation approaches that differ from those recommended in the WADA Technical Document for Identification Criteria (TD IDCR), including variations in the analytical thresholds, confirmation strategies, or validation approaches (53, 79, 80, 85). This heterogeneity likely reflects differences in study aims and regulatory contexts, as many of the included publications were not originally designed for anti-doping control (67, 68, 73). Nevertheless, the application of non-harmonized identification criteria may limit the comparability of findings and introduce uncertainty when results are interpreted within an anti-doping framework. Greater alignment with WADA TD IDCR recommendations would enhance analytical robustness, facilitate cross-study comparison, and improve the relevance of dietary supplement testing for athlete protection and risk assessment (52, 60, 69).

## 6 Limitations and future research

The findings of this review should be interpreted in light of several limitations. Considerable heterogeneity exists across the included studies in terms of study design, which limits direct comparability. Additionally, the review was limited to English-language publications, excluding book chapters, opinions, and conference articles, which may have excluded a few relevant studies; however, this restriction was necessary to manage the volume of identified records. Geographically, the evidence is disproportionately concentrated in the Americas, the Asia-Pacific region, and Europe, while research from the Middle East, North Africa (MENA), and South Asia is largely absent. Consequently, the findings may not be fully generalizable, and the true global prevalence of dietary supplement adulteration could be underestimated.

Future research should prioritize large-scale surveillance studies that systematically sample products from diverse markets (online and offline markets), particularly regions underrepresented in current literature. Other than technical advances, research on policy-driven and culturally grounded approaches is essential. Contextualized anti-doping education, stricter regulatory frameworks, and international cooperation will be key to safeguarding athletes' health and integrity, while ensuring that anti-doping practices remain globally harmonized and culturally resonant.

## 7 Conclusion

This systematic review demonstrates that inadvertent doping is a persistent risk due to the widespread contamination of dietary supplements with undeclared prohibited substances and pharmacological adulterants. Stimulants and anabolic-androgenic steroids were identified as the most prevalent contaminants, particularly in sports performance and weight-loss products, while the detection of SARMs, peptide hormones, and metabolic modulators points to the increasing pharmacological complexity of supplement adulteration. Regarding analytical detection, liquid chromatography–mass spectrometry was identified as the most comprehensive and sensitive approach, although complementary techniques, including GC-MS, spectroscopic methods, hybrid platforms, and immunoassays, play important roles in identifying specific substance classes. These findings indicate that no single analytical method is sufficient and highlight the necessity of integrated, multi-platform detection strategies to address the evolving and increasingly sophisticated landscape of supplement contamination.

Other than their technical implications, these findings carry important consequences for anti-doping regulation and athlete protection. The persistence of contamination across multiple supplement categories suggests that existing regulatory and surveillance mechanisms are insufficient to mitigate inadvertent anti-doping rule violations. Strengthening regulatory follow-up mechanisms, enhancing international cooperation in supplement testing, and improving the accessibility of reliable information for athletes and support personnel are therefore critical priorities. Future efforts should combine robust analytical capacity with relevant education and regulatory oversight to reduce vulnerabilities and better safeguard athlete health and integrity in a complex supplement marketplace.

## Data Availability

The original contributions presented in the study are included in the article/Supplementary Material, further inquiries can be directed to the corresponding author.
