# Supplementary material for: Systematic review of undeclared prohibited substances and pharmacological adulterants in dietary supplements: prevalence, detection, and risks in sport
Source: Front Sports Act Living. 2026 Mar 13;8:1740663. doi: 10.3389/fspor.2026.1740663 (PMC13021601; doi:10.3389/fspor.2026.1740663)
Supplement: Supplementary file 1 [file Table1.docx]

Supplementary Material

# Supplementary Data

**Table S1**: Summary of shortlisted studies highlighting adulteration in dietary supplements

| **SL** | **Study** | **Country** | **Type of supplement** | **Year** | **Study setting** | **Sample size** | **Adulterant analyzed** | **Detection level** | **Method of detection** | **Key findings** |
| --- | --- | --- | --- | --- | --- | --- | --- | --- | --- | --- |
| **1** | Wojtowicz et al. (2016) | Poland | OXPUMP Pre-Training Formula, Fruit Punch | 2015 | Lab-based | 3 volunteers’urine samples | N, N-dimethyl-2-phenylpropan-1-amine (NNDMPPA) | Urinary NN-DMPPA peaked at 189–303 ng/mL 2–3 hours post-ingestion and remained above 50 ng/mL for up to 22–23 hours. | GC-MS | NN-DMPPA was detectable in urine for up to 46 hours post-supplement ingestion using the GC-MS method. |
| **2** | Zhang et al. (2012) | USA | body-building supplements | 2010 | Lab-based | 13 dietary samples and 8 geranium oil samples | 1,3-Dimethylamylamine (DMAA) | DMAA concentrations in supplements ranged from ~0.1% to ~11%, while none was detected in the eight geranium oil samples. | HPLC-MS | GC and HPLC analyses suggest that DMAA in supplements is unlikely to be from geranium oil, as the extracted DMAA was racemic with diastereomeric ratios matching synthetic standards, and no DMAA ≥10 ppb was detected in the eight geranium oil samples. |
| **3** | Cohen et al. (2014) | USA | Pre-workout supplement | 2012 | Lab-based | 3 dietary samples | Methamphetamine analog N,α-diethyl-phenylethylamine (N,α-DEPEA). | Quantities exceeding 20 mg per serving indicated that the dietary supplement was contaminated with N,α-DEPEA. | UHPLC-LTQ Orbitrap XL MS And UHPLC-Q-TOF MS | The designer drug N,α-DEPEA, a structural analogue of methamphetamine, was detected in three lots of a widely available dietary supplement. |
| **4** | Russo et al. (2016) | Italy | Weight loss supplement | 2012 | Lab-based | 16 dietary samples | Fluoxetine, Triac (Tiratricol), Benfluorex, and Pseudoephedrine (PSE) | LOD values were 3.4 μg/mL (PSE), 1.1 μg/mL (Triac), 0.9 μg/mL (Fluoxetine), and 0.8 μg/mL (Benfluorex), with LOQs of 11.5, 3.7, 3.0, and 2.7 μg/mL, respectively; recoveries ranged from 75–78% at 20 μg/mL and 83–87% at 50 μg/mL, confirming reliable detection of all four contaminants. | Lc/Uv | Using a newly developed detection method, Fluoxetine was found in 31% of samples, Triac and PSE in 69%, and Benfluorex in 44%; co-occurrence included Triac + Fluoxetine (1 sample), Triac + PSE (3 samples), and all compounds (2 samples). |
| **5** | Abbate et al. (2015) | UK | Steroid-based dietary supplement | 2013 | Lab-based | 24 dietary products | DHEA (Dehydroepiandrosterone), Methyl-1-testosterone, Androstenedione, Androst-4-ene-3,11,17-trione, and Furazabol | Seventeen-alkylated anabolic steroids were detected at 3–31 mg per product, exceeding typical therapeutic doses of 2.5–20 mg/day. | GC-MS, Accurate-Mass LC-MS, HPLC-DAD, UV-Vis, And NMR. | Of 24 products tested, 23 contained steroids (including known anabolic agents), 16 had steroids differing from the label, and one product contained none. |
| **6** | Austin et al. (2014) | USA | Geraniaceae, geranium oil and dietary supplements | 2013 | Lab-based | 12 samples: One Pelargonium cultivar, one Geranium cultivar, | 1,3-Dimethylamylamine (DMAA) | No measurable levels of DMAA in Geranium, Pelargonium, or essential oils at a detection limit of 1–2 ng/g were present. Concentrations (weight%) of DMAA provided in Dietary samples ranged from 0.11% to 673%. | UPLC-MS/MS | This study indicates DMAA contained in dietary samples is of a synthetic origin and is not present in the plant species Geranium and Pelargonium; thus, the ‘natural’ origin and use of DMAA as an ingredient in DS is not substantiated. |
| **7** | Leaney et al. (2021) | UK | Sport dietary supplement | 2013 | Lab-based | 20 dietary samples | Selective androgen receptor modulators (SARMs) (Andarine, Ostarine, LGD-4033, RAD-140, YK-11, S23, ACP-105, and LGD-3303], Arimistane, ibutamoren, 1,3-dimethylamylamine (1,3-DMAA), etc. | In five products, higher amounts of these undeclared analytes were measured, ranging from approximately 0.6 mg/capsule to 6.8 mg/capsule | UHPLC-Q-Ex active HRAM MS | Only 6 of 20 supplements matched their labels; 60% contained undeclared analytes, with five products having higher amounts ranging from ~0.6 to 6.8 mg per capsule. |
| **8** | Cooper et al. (2018) | New Zealand | Sport dietary supplement | 2014 | Lab-based | 112 dietary sample | Androgens: Madol and Hemapolin, or selective androgen receptor modulator (SARM), 93746 | The androgen bioassay detected designer steroids (Madol, Hemapolin) and SARMs at 20 ng spiked into supplements, and could detect testosterone in oil-based but not powder matrices. | Yeast- And Mammalian Cell Androgen Bioassays | Out of 112 supplements, 6 showed strong androgenic activity in the yeast bioassay, with mammalian cell assays confirming this activity in 5 of them, indicating spiking or contamination with androgens. |
| **9** | Pascali et al. (2018) | Italy | Weight loss supplement (herbal tea extract and pills) | 2014 | Lab-based | 5 herbal tea extracts | Sibutramine, 4-hydroxyamphetamine, caffeine, and theophylline | Sibutramine was detected at 15 and 26 μg/mg (3.6 and 6.2 mg per unit), caffeine at 0.1–2.0% (5–100 mg per bag), and theophylline at 0.004–0.006% (0.2–0.3 mg per bag); all compounds were confirmed with precursor ions showing mass errors ≤10 ppm. | Q-TOF LC/MS) And LC-MS/MS | Q-TOF LC/MS screening combined with LC-MS/MS confirmation proved to be a rapid and effective method for detecting undeclared active compounds in commercial herbal teas. |
| **10** | Kim et al. (2022 a) | Korea | Steroid-based dietary supplement | 2022 | Lab-based | 35 samples | Arylpropionamides (3), Quinolinones (2), Pyrrolidinylbenzonitrile (1), Indoles (2), Tropanols (2), Phenylaxadiazole (1), Hydantoins (2), Phenylthiazole (1), Nitrothiophene (1), And Steroidal Derivatives (20). | Sibutramine was detected at 15–26 μg/mg (3.6–6.2 mg/unit), caffeine at 0.1–2.0% (5–100 mg/bag), and theophylline at 0.004–0.006% (0.2–0.3 mg/bag), all confirmed with precursor ion mass errors ≤10 ppm. | LC-Q-TOF MS | Prohibited SARMs and steroids were detected in seized muscle-building supplements, with 10 of 35 samples containing one or more SARMs, including MK-677, RAD-140, Ostarine, LGD-4033, GW-501516, Andarine, S-23, SR-9009, YK-11, and hydrolyzed YK-11. |
| **11** | Kim et al. (2022 b) | Korea | Steroid-based dietary supplement | 2022 | Lab-based | 75 real dietary supplements and seized samples | Osterine, Andarine, MK-667, LGD-4033, YK-11, SR-9009, GW-501516, and RAD-140 | ChatGPT said:  Adulterant concentrations in seized capsules ranged from 2.14 μg/g (LGD-4033) to 71,989 μg/g (Andarine). | LC–ESI–MS/MS And  UHPLC‑PDA | Screening 18 compounds, 10 SARMs (including MK-677, YK-11) were detected in 25 seized capsules. |
| **12** | Cheng et al. (2017) | China | Weight loss supplement | 2014 | Lab-based | 120 dietary herbal weight loss samples | Sibutramine and Fluoxetine | Of 120 weight-loss supplement batches, 29 tested positive for sibutramine, fluoxetine, or both, with sibutramine being the most frequent and exceeding 100 mg/g. | UHPLC-LTQ-Orbitrap HRMS | The UHPLC-LTQ-Orbitrap HRMS method was developed and validated for detecting illegal weight-loss drugs, with 29 of 120 plant supplement batches testing positive for sibutramine, fluoxetine, or both. |
| **13** | dos Santos et al. (2018) | USA | Sport dietary supplement | 2015 | Lab-based | 108 samples | 1,3-Dimethylamylamine Along With Sibutramine And Methylphenidate |  | DART-MS/MS | DMAA was detected in 20% of seized supplements, often with sibutramine and caffeine; of 108 samples, ~50% contained sibutramine and 10% methylphenidate. |
| **14** | Zhao et al. (2018) | USA | Sport Dietary supplement, Weight loss supplement | 2015 | Lab-based | 32 samples | Phenethylamines (Peas) Phenethylamine, Synephrine, Oxilofrine, Hordenine, Β-Methylphenethylamine, N-Methyltyramine, Octopamine, Deterenol | Phenethylamine was detected in 16 of 32 products, with concentrations ranging from 0.7% to 29.9%. | NMR Method | NMR reliably identified and quantified phenethylamine in dietary supplements, with adulteration found in 16 of 32 products, including four that did not list it on the label. |
| **15** | Helmlin et al. (2016) | Germany | Diuretics: Non-steroidal anti-inflammatory tablet contaminated with HCTZ | 2015 | lab-based | Athlete’s urine sample and diuretics Tabet strip | Hydrochlorothiazide (HCTZ, 6-chloro-3,4-dihydro-2H-1,2,4-benzothiadiazine-7-sulfonamide-1,1-diox- ide) | Urine tests showed HCTZ concentrations of 1–16 ng/mL after ingestion of contaminated NSAID tablets. | LC-MS/MS | Ingestion of ibuprofen tablets was identified as the source of the prohibited diuretic HCTZ found in an athlete’s urine sample. |
| **16** | Roiffé et al. (2019) | Brazil | Whey Protein Food Supplement (WPFS) | 2019 | Lab-based | 11 Whey protein food supplement samples | 105 substances: anabolic agents, beta-agonists, hormone and metabolic modulators, diuretics, and stimulants, anorectic agents |  | LC-Orbitrap HRMS | LC-Orbitrap HRMS successfully detected adulteration in seven of 11 commercial samples with diuretics (conivaptan, polythiazide) and the stimulant benfluorex. |
| **17** | Wójtowicz et al. (2015) | Poland | Sport dietary supplement | 2016 | Lab-based | 3 healthy volunteers’ urine samples | 2-ethylamino-1-phenylbutane (EAPB) and its metabolite, 2-amino-1-phenylbutane (APB) | EAPB was found in 14/17 unlabeled supplements (1.8–16.1 mg/g), with urinary levels of 2.2–4.2 μg/mL for EAPB and 1.1–5.1 μg/mL for its metabolite APB after oral administration. | GC-MS | Quantitative analysis of the designer doping agent EAPB and its metabolite APB in urine, as well as EAPB in 14 /17 unlabeled dietary supplements, was conducted. |
| **18** | Stajić et al. (2017) | Serbia | Weight loss supplement | 2016 | Lab-based | 19 dietary samples | Higenamine | Higenamine concentrations in two adulterated samples were 6.42 ng/mL and 18.93 ng/mg. | UPLC/HILIC-/MS/MS | The validated UPLC/HILIC-MS/MS method identified and quantified higenamine in dietary supplements, with 2 of 19 samples found adulterated. |
| **19** | Kozhuharov et al. (2023) | Bulgaria | Sport dietary supplement | 2023 | Lab-based | 50 dietary samples | Sibutramine | The LOD and LOQ for sibutramine were 0.181 μg/mL and 0.549 μg/mL, respectively; analysis of 50 samples found six containing varying amounts of sibutramine. | GC-MS | The GC-MS method effectively detected sibutramine contamination, with 6 of 50 dietary supplements found to be contaminated. |
| **20** | Zaharieva et al. (2023) | Bulgaria | Steroid-based dietary supplement | 2023 | Lab-based | 9 dietary samples | Steroid Structure Substance: Testosterone, Testosterone Propionate, Testosterone Enanthate, etc. |  | HPLC – diode array detector | An HPLC-DAD method was developed and validated for the simultaneous determination of 13 steroid compounds in dietary supplements for sports. |
| **21** | Esposito et al. (2023) | Italy | Weight loss supplement, energy booster | 2023 | Lab-based | 91 dietary samples | Alkaloids and biogenic amines detected included caffeine, synephrine, agmatine sulfate, yohimbine, phenethylamine, and icariin. |  | LC-MS/MS Method | Analysis revealed 26% of samples were mislabeled, underscoring frequent fraud in natural supplements; LC-MS/MS offers a reliable tool for consumer protection. |
| **22** | Schreiber et al. (2024) | Japan | Steroid-based dietary supplement | 2024 | Lab-based | 15 dietary samples | Anabolic-androgenic steroids (methandienone) |  | Ultra‑High-Performance Liquid Chromatography–Fourier Transform Mass Spectrometry | Analysis revealed that of 15 samples, 6.7% were confirmed falsified, 80% were falsified or unlicensed, and 13.3% lacked manufacturer or labeling information. |
| **23** | Thevis et al. (2015) | Germany | diuretics: anti-malarial drug | 2015 | Case study | urine samples of 3 athletes | Chlorazanil |  | LC-HRMS/MS | The study shows that chlorazanil is not a direct metabolite of proguanil but can form artificially from its metabolite N-(4-chlorophenyl)-biguanide under specific urinary conditions, such as elevated formaldehyde. This explains why athletes using proguanil for malaria prophylaxis may test positive for the banned diuretic chlorazanil in anti-doping controls. |
| **24** | Thomas et al. (2010) | Germany | Sport dietary supplement | 2010 | Case study | Supplement Tablets with GHRP-2 | Growth hormone-releasing peptide (GHRP-2) | The content of GHRP-2 was determined with approximately 50 μg per tablet. | LC–QQQ-MS | The study identified and quantified the prohibited peptide GHRP-2 in tablets marketed as nutritional supplements, confirming its presence in black-market products. Analytical data from LC–HRMS and LC–MS/MS provide a basis for developing routine anti-doping assays to detect GHRP-2 misuse in athletes. |
| **25** | Holubová et al. (2019) | Czech republic | Steroid-based dietary supplement | 2019 | Lab-based | 3 dietary samples | Anabolic-androgenic steroids (Mesterolone) |  | Enzyme-Linked Immunosorbent Assay (ELISA) And Lateral Flow Immunoassay (LFIA) | Two new immunochemical methods (ELISA and LFIA) were developed for sensitive and specific detection of the anabolic steroid Mesterolone in food supplements, showing good correlation with UHPLC–MS/MS. The LFIA method proved suitable for rapid screening without cleanup, supporting its use in detecting steroid adulteration in dietary supplements. |
| **26** | Petkova-Gueorguieva et al. (2023) | Bulgaria | Steroid-based dietary supplement | 2023 | Lab-based | 24 dietary products | Methandienone, Methandienone, Methyltestosterone, Oxandrolone, Methandienone, Methyltestosterone, Methandienone, Oxandrolone, Methandienone, Stanozolol, Metenolone |  | LC Method | More than half of the analyzed food supplements (12/23) contained undeclared anabolic steroids, some with multiple banned substances per sample. The findings confirm hidden doping risks, as product labels concealed these ingredients while falsely claiming safety and efficacy. |
| **27** | Strano-Rossi et al. (2015) | Italy | Sports dietary supplement | 2015 | Lab-based | 36 dietary supplements | Stimulants (Ephedrines, Caffeine, Anorectic Drugs Such as Phentermine, Phendimetrazine, Phenmetrazine, Fenfluramine, Benfluorex, Mephentermine, Fen-Canfamine, Sibutramine) And PDE5I (Sildenafil, Vardenafil and Tadalafil) | Detected ephedrine at 370–1000 ng/g, ephedrine/pseudoephedrine at 460–540 ng/g, caffeine at 225–40,500 ng/g, and sildenafil in “Kamagra” tablets at ~200 mg/g (~100 mg per tablet). Some compounds were undeclared on the product labels. | LC–HRMS | Analysis of 36 dietary supplements detected ephedrine/pseudoephedrine in 4, caffeine in 8, and sildenafil in 4 samples, with some undeclared on labels. |
| **28** | Shin et al. (2020) | Korea | Sport dietary supplement | 2020 | Lab-based | 200 dietary supplements | Many illicit compounds | The concentrations of illicit compounds detected in dietary supplements ranged from 0.51 to 226 mg/g. | LC-MS/MS | The method detected illicit compounds in 13.5% of dietary supplements (27/200), with concentrations ranging from 0.51 to 226 mg/g. This approach is effective for monitoring adulteration in supplements. |
| **29** | Favretto et al. (2019) | Italy | Diuretics | 2019 | Case study | 3 healthy volunteers’ urine samples | Diuretics: Hydrochlorothiazide | Post-administration specimens were found to contain HCTZ at concentrations of 5–230 ng/mL, which supported the accidental inadvertent intake of the prohibited substance by the athlete. | UPLC-MS/MS | Analysis confirmed that the athlete’s nutritional supplements contained hydrochlorothiazide (HCTZ) at μg/g levels, explaining the low urinary concentrations (<10 ng/mL). Controlled ingestion by volunteers produced similar urinary HCTZ levels (5–230 ng/mL), supporting accidental intake as the cause of the AAFs. |
| **30** | Kazlauskas et al. (2015) | Poland | Sport dietary supplement | 2015 | Case study | urine samples of 4 athletes | Stimulant: N, N-dimethyl-2-phenylpropan-1-amine | The measured concentrations of NN-DMPPA were between 0.51 and 6.51 μg/mL in athletes’ urine samples. | GC-MS | Urine samples from four athletes contained the banned stimulant NN-DMPPA at 0.51–6.51 μg/mL, confirmed by GC–MS. Analysis of the supplement NOXPUMP revealed undeclared NN-DMPPA (121.7 μg/g) along with β-methylphenethylamine, highlighting hidden doping risks. |
| **31** | Cohen et al. (2021) | USA | Weightloss supplement | 2021 | Lab-based | 17 dietary supplements | Stimulant: Deterenol, Phenpromethamine, Oxilofrine, Octodrine, Beta-Methylphenylethylamine (1,3-DMBA), and Higenamine, Dimethylamylamine (1,4-DMAA), 1,3-Dimethylbutylamine (BMPEA), etc. | The detected amounts of nine stimulants per recommended serving varied widely, including 2.7–17 mg of Deterenol, 1.3–20 mg of Phenpromethamine, 5.7–92 mg of beta-Methylphenylethylamine, 18–73 mg of octodrine, 18–55 mg of Oxilofrine, 48 mg of Higenamine, 17 mg of 1,3-dimethylamylamine, 1.8–6.6 mg of 1,3-dimethylbutylamine, and 5.3 mg of 1,4-dimethylamylamine | UHPLC Quadrupole-Orbitrap Mass Spectrometry | Seventeen supplement brands were analyzed, with several containing multiple prohibited stimulants: 4 brands (24%) had 2 stimulants, 2 brands (12%) contained 3 stimulants, and 2 brands (12%) included 4 stimulants in a single product. |
| **32** | Baglietto et al. (2024) | Italy | Sports dietary supplement | 2024 | Lab-based | 8 dietary supplements | Polar compounds: diuretics, stimulants, β2-agonists, methylxanthines, and sweeteners. | Four methylxanthines and two artificial sweeteners were detected and quantified at levels ranging from 0.02 to 192 mg/g, with some deviations from the amounts declared on the product labels. | HILIC‑MS/MS | The developed method achieved recoveries of 40–107% and precision ≤11%, detecting four methylxanthines and two artificial sweeteners in real samples at varying concentrations. Additionally, MS/MS scans revealed a clenbuterol-like signal in one supplement, indicating potential undeclared β2-agonist presence. |
| **33** | Monakhova et al. (2014) | Germany | Sport dietary supplement | 2014 | Lab-based | 16 dietary supplements | 1,3-dimethylamylamine (DMAA) | The detection concentrations of DMAA-HCl in the analyzed products ranged from 3.1 g/kg to 415 g/kg. | NMR | The NMR method detected DMAA-HCl in 9 out of 16 products, with concentrations ranging from 3.1 to 415 g/kg. This approach is suitable for routine testing in food, customs, and anti-doping laboratories. |
| **34** | Jendrzejewska et al. (2025) | Poland | Steroid-based dietary supplement | 2025 | Lab-based | 16 dietary supplements | SARMs: Andarine, Ligandrol, Ostarine, and Testolone |  | Raman Spectroscopy, Differential Scanning Calorimetry, Thermogravimetry, And X-Ray Powder Diffraction | Using rapid analytical methods, including Raman spectroscopy, SARMs were confirmed in 9 of 16 samples, while Testolone, Ostarine, and Andarine were not reliably detected in the remaining 7 samples. |
| **35** | Lee et al. (2021) | Korea | Diuretics in fat burner | 2021 | Lab-based | 23 dietary supplements | Thiazide diuretics (e.g., Bendroflumethiazide), loop diuretics (e.g., Bumetanide), potassium-sparing diuretics (e.g., Amiloride), and Carbonic anhydrase inhibitors (e.g., Acetazolamide) | Approximately 5% of the samples were found to be illegally contaminated with diuretics at a concentration of 0.051–162 mg/g. | UHPLC-Q-Orbitrap | Screening of 124 products using UHPLC-Q-Orbitrap (LC-HRMS) revealed that about 5% were contaminated with diuretics at 0.051–162 mg/g. |
| **36** | Lee et al. (2020) | Korea | Steroid-based dietary supplement | 2020 | Lab-based | 198 dietary supplements | 28 different anabolic-androgenic steroids | Among the 198 samples analyzed, two were found to contain testosterone and stanozolol at concentrations of 0.27 μg/g and 0.023 μg/g, respectively. | Quechers Extraction followed by Liquid Chromatography-Tandem Mass Spectrometry | Among 198 protein supplements collected from various markets, two contained testosterone and stanozolol, while 5α-hydroxylaxogenin was detected for the first time in three overseas-purchased products. A modified QuEChERS method was successfully applied for screening and quantifying anabolic steroids in these supplements. |
| **37** | Ramos et al. (2021) | Brazil | Weightloss supplement | 2021 | Lab-based | 5 dietary supplements | Fluoxetine |  | Voltametric Method | An electrochemical method using boron-doped diamond electrodes was developed for rapid screening and quantification of fluoxetine in weight loss supplements and herbal medicines. |
| **38** | Saadabadi et al. (2022) | Iran | Steroid-based dietary supplement | 2022 | Lab-based | 50 dietary supplements | anabolic-androgenic steroids (AAS): 19-Nor Androstenedione, 19-Nortestosterone, Methyl Testosterone, Clostebol, etc, | AAS was not detected in the samples | HPTLC | A validated HPTLC method was developed for the simultaneous detection of ten anabolic steroids in sports supplements, showing good precision, accuracy, and sensitivity. Analysis of 50 real supplement samples revealed no detectable steroid contamination. |
| **39** | Kim et al. (2025) | Korea | dietary supplement for respiratory function | 2025 | Lab-based | 52 dietary supplements | Respiratory drug: Theobromine, Theophylline, Dropropizine, Guaifenesin, Tramazoline, Azelastine, Fexofenadine, etc. | One sample was found to have a theobromine content of 313.7 mg/g. | LC–ESI–MS/MS And LC–QTOF-MS. | A validated LC–ESI–MS/MS method was developed for the simultaneous detection of 11 respiratory drug substances in dietary supplements. Application to 52 products revealed one sample containing 313.7 mg/g of theobromine. |
| **40** | Alaedini et al. (2021) | Iran | Steroid-based dietary supplement | 2021 | Lab-based | 30 dietary supplements | androgenic anabolic steroids (AAS): methyltestosterone and 4-androstenedione | 4-Androstenedione was detected in 11 samples, comprising 9 whey products at 1.578 ± 0.154 ng/g and 2 whey albumin samples at 1.134 ng/g and 1.474 ng/g. | UPLC-MS/MS | Analysis of sports supplements using UPLC-MS/MS revealed undeclared anabolic hormones, with 4-androstenedione detected in 11 samples, highlighting potential health risks for athletes. |
| **41** | Cohen et al. (2019) | USA | Weight loss supplement | 2019 | Lab-based | 24 dietary supplements | Higenamine | Dietary supplements sold in the United States were found to contain up to 62 ± 6.0 mg of the stimulant Higenamine per serving. | UHPLC-MS/MS And UHPLC-QTOF-MS. | Analysis of 24 supplements revealed Higenamine levels ranging from trace amounts to 62 ± 6.0 mg per serving. Labeling was inaccurate in five products, with actual Higenamine content varying from <0.01% to 200% of the declared amount. |
| **42** | Thevis et al. (2013) | Germany | Traditional Chinese Medicine | 2013 | Case study | 27 urine samples of 5 athletes | Steroidal in musk samples: 5α-Androstane-3,17-dione, 5β-Androstane-3,17-dione, 3α-Hydroxy-5α-androstan-17-one, etc. |  | Isotope-Ratio Mass Spectrometry (IRMS) | Musk pod extracts contain natural steroids that can alter athletes’ steroid profiles. Their use by five female athletes led to confirmed doping violations via IRMS analysis. |
| **43** | Duiven et al. (2021) | Netherlands | Sport dietary supplement | 2021 | Lab-based | 66 dietary supplements | Anabolic agents, Stimulants, Beta-2 agonists, Beta-blockers |  | GC-MS And LC-MS | Out of 66 products, 25 (38%) contained undeclared doping substances, including the stimulants Oxilofrine, β-methylphenethylamine, N,β-dimethylphenethylamine, 4-methylhexan-2-amine (Methylhexaneamine, 1,3-dimethylamylamine, the anabolic steroids Boldione (1,4-androstadiene-3,17-dione) and 5-androstene-3β,17α-diol (17α-AED), the beta-2 agonist Higenamine, and the beta-blocker bisoprolol. |
